# Relationship between Serum Uric Acid Levels and Chronic Kidney Disease in a Japanese Cohort with Normal or Mildly Reduced Kidney Function

**DOI:** 10.1371/journal.pone.0137449

**Published:** 2015-09-10

**Authors:** Tadashi Toyama, Kengo Furuichi, Miho Shimizu, Akinori Hara, Yasunori Iwata, Norihiko Sakai, Vlado Perkovic, Makoto Kobayashi, Toshiki Mano, Shuichi Kaneko, Takashi Wada

**Affiliations:** 1 Division of Nephrology, Kanazawa University Hospital, Kanazawa, Japan; 2 The George Institute for Global Health, University of Sydney, Sydney, NSW, Australia; 3 Tama Graduate School of Management and Information Science, Tokyo, Japan; 4 CRECON Research and Consulting Inc., Tokyo, Japan; 5 Department of Disease Control and Homeostasis, Institute of Medical, Pharmaceutical and Health Sciences, Kanazawa University, Kanazawa, Japan; 6 Department of Laboratory Medicine, Institute of Medical, Pharmaceutical and Health Sciences, Kanazawa University, Kanazawa, Japan; University Medical Center Utrecht, NETHERLANDS

## Abstract

**Background:**

Some observational studies have shown the relationships between hyperuricemia and chronic kidney disease (CKD); however, the threshold of serum uric acid (SUA) for deterioration of kidney function and the association between SUA and kidney injury by baseline kidney function remains unclear. This study aimed to clarify the relationships between SUA and reduced kidney function.

**Methods:**

We analyzed a historical cohort of male Japanese individuals who underwent medical checkup between 1998 and 2007. Participants with baseline data and who were followed up for at least one year were included and stratified according to baseline kidney function. Kidney function was classified as normal [estimated glomerular filtration rate (eGFR) ≥ 90 ml/min/1.73 m^2^] or mildly reduced (eGFR 60–89 ml/min/1.73 m^2^). The outcome measured was kidney impairment defined as a decrease in eGFR to < 60 ml/min/1.73 m^2^. Associations between SUA and risk for outcome and eGFR slopes were assessed.

**Results:**

A total of 41632 subjects with mean age 45.4 years were included. During a mean follow-up of four years, 3186 (7.6%) subjects developed kidney dysfunction. Subjects with SUA ≥ 6.0 mg/dL had a significantly increased risk for kidney impairment compared with subjects with SUA of 4–4.9 mg/dL. SUA threshold levels were different according to baseline kidney function; SUA ≥ 7.0 and ≥ 6.0 mg/dL for normal and mildly reduced kidney function, respectively. Approximately the same trends were observed for eGFR slopes.

**Conclusion:**

In the general population, hyperuricemia appears to be a risk factor for kidney impairment in males. For participants with mild kidney dysfunction, even a slight elevation of SUA can be a risk factor.

## Introduction

Chronic kidney disease (CKD) is a known risk factor, not only for end-stage kidney diseases, but also for cardiovascular mortality and all-cause mortality.[1] Globally, the number of deaths from CKD has increased by more than 80% over the past 20 years.[2] Many factors, including low estimated glomerular filtration rate (eGFR), elevated proteinuria, diabetes, and hypertension have been identified as risk factors for the development and progression of CKD.[1] Recently, serum uric acid (SUA) was proposed as a potential risk factor for new onset of kidney disease in the general population [3, 4] From the pathophysiological perspective, hyperuricemia results in the progression of renal dysfunction through preglomerular arteriolopathy characterized by hyalinosis and wall thickening [5]; a meta-analysis suggested that allopurinol therapy retarded the progression of CKD.[6] However, the threshold for the risk of CKD and differences in gender are still remain uncertain.

On the other hand, a very low uric acid level was thought to be a risk factor for acute kidney injury.[7] In fact, hypouricemia was thought to be a candidate risk factor for CKD [4]; however, the precise impact is still uncertain. A study on type 1 diabetic patients demonstrated that risks for CKD stage 3 linearly increased with SUA levels across the normal range including SUA < 3.0 mg/dL.[8] For all-cause mortality, J-shape relationships between uric acid levels and risks were observed in a previous study.[9]

In the present CKD guidelines, eGFR of 60–89 ml/min/1.73 m^2^ without proteinuria is not classified as CKD because the risks for kidney failure and cardiovascular disease are low in this group. However, recent studies revealed mild kidney dysfunction combined with specific risk factors, such as chest pain [10] and mineral bone disorder [11], as probable risk factors.

To date, the threshold of uric acid as a risk factor for CKD by baseline kidney function and the impact of low uric acid are not revealed. Precise understanding of these is necessary for treatment and prevention of CKD.

Therefore, with the availability of a large Japanese cohort, we aimed to clarify the precise relationship between uric acid levels and development of CKD, and to elucidate the effects of hypouricemia.

## Methods

### Subjects

We conducted a historic cohort study among individuals recruited from a Japanese general population who underwent an annual medical checkup from 1998 to 2007 in Kanagawa Prefecture. subjects were included if they were followed up for at least one year. Data regarding SUA levels, serum creatinine, urine protein (dipstick measurement), hemoglobin, blood pressure, HbA1c, fasting plasma glucose high-density lipoprotein (HDL), triglycerides, and body mass index (BMI) were recorded. Females were not included in the analysis because the number of participants with hyperuricemia was limited in the study population. Serum creatinine was recorded at baseline and at least once during follow-up. Subjects aged < 18 years of age or those with baseline eGFR < 60 ml/min/1.73 m^2^ were excluded from analysis.

### Measurement of study variables

Blood pressure was measured at rest in the sitting position. Blood samples were collected in the fasting state. Urine dipstick analysis was performed using random spot urine samples. Serum creatinine values were measured by the enzymatic method, and eGFR was calculated from serum creatinine using equations developed by the Japanese Society of Nephrology.[12] The value for HbA1c (%) is estimated as an NGSP (National Glycohemoglobin Standardization Program) equivalent value (%) calculated by the formula HbA1c (%) = HbA1c (JDS) (%) + 0.4%, considering the relational expression of HbA1c (JDS) (%) measured by the previous Japanese standard substance and measurement methods and HbA1c (NGSP).[13] Diabetes was defined as HbA1c ≥ 6.5% (NGSP) and/or fasting plasma glucose ≥ 126 mg/dL.

### Definition of outcomes

Kidney dysfunction was defined as a decrease in eGFR to < 60 ml/min/1.73 m^2^ during follow-up. Participants were followed from their first analysis of eGFR until 2007 or their last analysis of eGFR.

Each patient’s annual changes in eGFR were calculated by least-square method using all measurements of eGFR. For obtaining accurate eGFR slope, it has been suggested that 1- to 2-year observation period may not accurately reflect the true rate of decline.[14] According to the suggestion, subjects who were followed at least three years were included for analysis of slope.

### Statistical analysis

Subject were stratified into five groups according to the uric acid level (< 4, 4.0–4.9, 5.0–5.9, 6.0–6.9, and ≥ 7.0 mg/dL). Baseline data with normal distribution were reported as the mean (± standard deviation), categorical numbers as proportions. Dipstick test results were divided into three categories: negative/trace, 1+, and ≥ 2+. Event rates per 1000 person-years were calculated for the follow-up period by dividing the number of events by the number of person-years of follow-up. Cox-proportional hazards model with 95% confidence interval was used to estimate risks for kidney dysfunction according to uric acid levels. The proportional hazard assumption was tested using plots of the log-log survival curves. Multivariate-adjusted mean values of eGFR slopes were calculated by covariance analysis and were compared according to SUA levels.

For analyses, SUA level of 4.0–4.9 mg/dL was defined as the reference value for other categories. Adjustment for baseline age, eGFR level, systolic blood pressure, BMI, hemoglobin, HDL, triglyceride, urinary protein, and diabetes at baseline was applied in the multivariate analyses. Adjusted confounding factors were selected in consideration of a previous report on changes in GFR.[15] The p-value for trend was calculated by including SUA category as a continuous variable in the model. A two-tailed significance level of 0.05 was used in all tests. All analyses were performed using Stata/IC statistical software (version 12.1; StataCorp LP, College Station, TX, USA).

### Ethics statement

The study protocol was approved by the ethics committee of Kanazawa University (approval number: 942). All analyses were performed using de-identified data.

## Results

### Baseline characteristics of subjects

**Fig 1:**
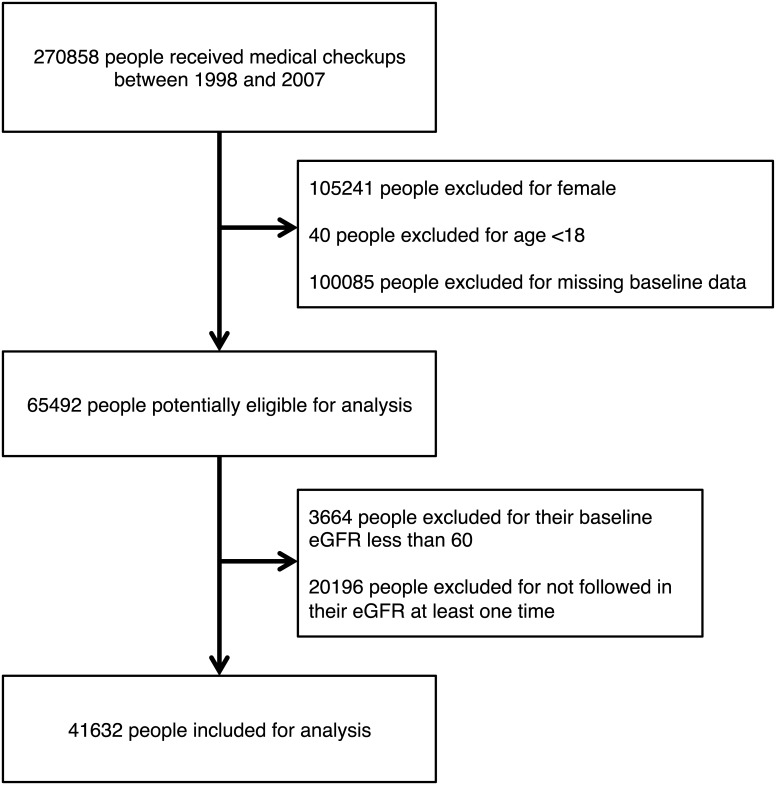
Flow diagram for subject selection. Baseline variables contain laboratory results (serum uric acid, serum creatinine, dipstick measured urine proteins, hemoglobin, high-density lipoprotein, triglycerides) physical examination (body mass index, systolic blood pressure, and diastolic blood pressure), and diabetes.

A flow diagram describing the selection of the study subjects is presented in Fig 1. Of 270858 subjects who underwent a medical checkup between 1998 and 2007, 41632 met the inclusion criteria and were selected for the present analysis.

Table 1 shows the baseline characteristics of the subjects according to uric acid level. Mean serum uric acid level was 6.0 mg/dL. Mean follow-up period was 4.0 (SD 2.7) years, and mean age was 45.4 yrs. SUA level in the range of 5.0–5.9 mg/dL was predominant. Proteinuria, hemoglobin, level of blood pressure, triglycerides, BMI, and prevalence of diabetes increased with higher uric acid levels; eGFR and HDL cholesterol levels decreased with higher uric acid levels. Mean baseline age was lower in higher uric acid levels. Majority (97.2%) of subjects had dipstick negative/trace proteinuria, and most patients (99.6%) had hemoglobin levels of ≥ 12.0 g/dL.

**Table 1:** Baseline characteristics of subjects according to baseline uric acid levels and gender.

		Serum Uric Acid (mg/dL)					
	All	<4.0	4.0–4.9	5.0–5.9	6.0–6.9	≥ 7.0	P for trend
N	41632	1899	5644	12668	12132	9109
Serum uric acid, mg/dL	6.0 (1.3)	3.4 (0.6)	4.5 (0.3)	5.5 (0.3)	6.4 (0.3)	7.7 (0.7)	
Age, years	45.4 (11.9)	47.9 (12.3)	47.3 (12.5)	45.7 (12.1)	44.9 (11.8)	44.3 (10.9)	<0.01
eGFR, ml/min/1.73 m^2^	84.3 (15.4)	88.9 (18.7)	87.6 (16.6)	85.5 (16.1)	83.3 (14.2)	81.0 (13.8)	<0.01
Proteinuria, number (%)	1175 (2.8)	54 (2.8)	134 (2.4)	284 (2.2)	337 (2.7)	366 (4.0)	<0.01
Hb, g/dL	15.1 (1.0)	14.9 (1.1)	14.9 (1.1)	15.1 (1.0)	15.2 (1.0)	15.3 (1.0)	<0.01
Systolic blood pressure, mmHg	125.3 (16.1)	123.2 (16.3)	123.3 (15.9)	123.8 (15.6)	125.4 (15.9)	128.9 (16.3)	<0.01
Diastolic blood pressure, mmHg	77.9 (11.0)	76.0 (11.0)	76.1 (10.6)	76.7 (10.6)	78.2 (10.9)	80.8 (11.1)	<0.01
HDL cholesterol, mg/dL	55.4 (14.0)	57.5 (14.5)	56.7 (14.2)	56.2 (14.1)	55.0 (13.7)	53.6 (13.6)	<0.01
Triglycerides, mg/dL	131.6 (109.5)	109.2 (87.1)	111.6 (92.8)	117.6 (90.8)	132.6 (105.4)	166.8 (139.7)	<0.01
BMI, kg/m^2^	23.5 (3.2)	22.5 (2.9)	22.5 (3.0)	22.9 (2.9)	23.6 (3.0)	24.8 (3.4)	<0.01
Diabetes, number (%)	1175 (2.8)	54 (2.8)	134 (2.4)	284 (2.2)	337 (2.7)	366 (4.0)	<0.01
HbA1c (NGSP), % (n = 29911)	5.6 (0.7)	5.8 (1.3)	5.7 (1.0)	5.6 (0.7)	5.5 (0.6)	5.5 (0.6)	<0.01
Fasting plasma glucose, mg/dL (n = 37238)	96.9 (22.1)	102.9 (36.4)	99.2 (30.0)	96.2 (21.9)	95.7 (18.2)	96.7 (16.8)	<0.01

Data are presented as numbers (%), means (SD). Totals do not always add to 100% because of rounding.

Abbreviations: NGSP, National Glycohemoglobin Standardization Program; n, number of participants with values.

### Uric acid and risk for CKD

During the follow-up period, 7.6% of patients developed kidney dysfunction. Number, person-years of follow-up, and hazard ratio of events are presented in Table 2.

**Table 2:** Adjusted hazard ratios for risk of renal dysfunction according to quartiles of baseline serum uric acid.

	Serum Uric Acid (mg/dL)					
	<4.0	4.0–4.9	5.0–5.9	6.0–6.9	≥ 7.0	P for trend
Number of events	124	325	824	1032	881
Person-years	7879	23112	51303	48923	35428
Events per 1000 person-years	15.7	14.1	16.1	22.1	24.9
Age-adjusted HR (95%CI)	1.09 (0.89—1.34)	1 (reference)	1.29 (1.14—1.47)	1.85 (1.63—2.10)	2.34 (2.06—2.66)	<0.01
Multivariate-adjusted HR (95%CI)*	1.10 (0.90—1.35)	1 (reference)	1.07 (0.94—1.22)	1.24 (1.09—1.41)	1.23 (1.08—1.41)	<0.01

* Adjusted for baseline age, eGFR, systolic blood pressure, body mass index, hemoglobin, HDL cholesterol, triglyceride, urinary protein, and diabetes.

**Fig 2:**
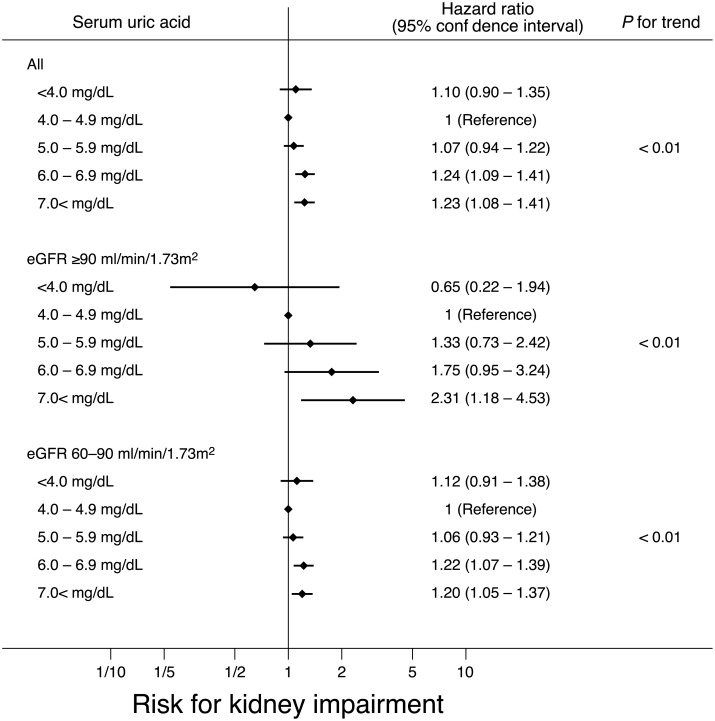
Relationships of uric acid and kidney dysfunction and baseline kidney function. Adjusted for baseline age, eGFR, systolic blood pressure, body mass index, hemoglobin, HDL cholesterol, triglyceride, urinary protein, and diabetes.

Higher rates of CKD events were observed in the presence of higher uric acid levels. However, in the multivariate adjusted hazard ratio, trends of higher hazard ratio for higher SUA levels was only significant. We also assessed the risk for CKD according to baseline kidney function (Fig 2).

In participants with mild kidney dysfunction, using SUA level 4.0–4.9 mg/dL as reference, SUA level 6.0–6.9 mg/dL was associated with significant risk (hazard ratio, 1.22; 95% confidence interval [CI], 1.07–1.39); however, the hazard ratio was not significant in participants with normal kidney function (hazard ratio, 1.75; 95% CI, 0.95–3.24). In both subgroups of baseline eGFR, risks for CKD was significantly higher with higher SUA levels. Trend almost linearly increased in logarithmic scale in the subgroup with eGFR ≥ 90 ml/min/1.73 m^2^.

Based on Table 1, SUA was significantly associated with baseline eGFR. We conducted a more stratified analysis by varying baseline eGFRs (60–74, 75–89, 90–104, and ≥ 105 ml/min/1.73 m^2^) without adjustment for baseline eGFR (S1 Fig). The result was approximately the same, although the associations of hyperuricemia were not observed in patients with baseline eGFR of ≥ 105 ml/min/1.73 m^2^.

Throughout analyses by baseline eGFR, participants with low SUA had no significant risk for CKD. Positive risk of SUA level < 4.0 mg/dL (hazard ratio 1.12; 95% CI, 0.91—1.38) was observed solely in subjects with mild kidney dysfunction.

### Uric acid and rate of decline in eGFR

**Fig 3:**
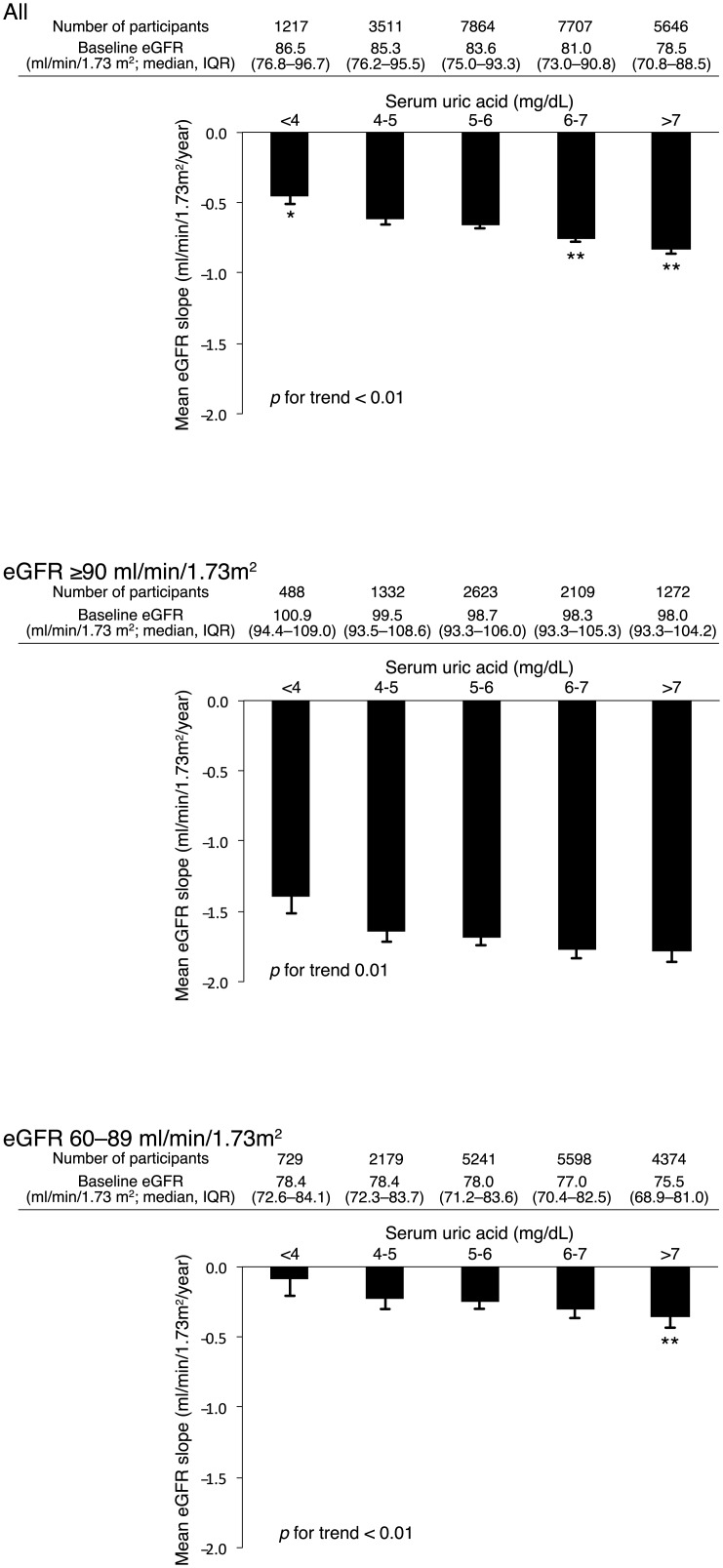
Association between uric acid and eGFR slope according to baseline kidney function. Multivariate-adjusted mean values of eGFR slope adjusted for baseline age, eGFR, systolic blood pressure, body mass index, hemoglobin, HDL cholesterol, triglyceride, urinary protein, and diabetes. Values are shown as mean ± SE. * P < 0.05, ** P < 0.01 compared with baseline serum uric acid 4.0–4.9 mg/dL.

Associations between changes in eGFR and SUA levels were examined using multivariate-adjusted mean values of eGFR slope. As with the risks for CKD, SUA ≥ 6.0 mg/dL was a significant risk factor for rapid decline in eGFR (Fig 3) and these findings were mainly observed in patients with mild kidney dysfunction (p < 0.01 for SUA ≥ 7.0 mg/dL).

## Discussion

This cohort study aimed to determine the threshold SUA level for CKD risk and to confirm J-shape relationships in a Japanese general population. We found significant trend towards CKD risk according to higher SUA levels; based on of the significant-levels of SUA in hazard ratios for risk of eGFR < 60 ml/min/1.73 m^2^ and slope of eGFR, threshold SUA levels was 7.0 mg/dL for eGFR ≥ 90 ml/min/1.73 m^2^ and 6.0 mg/dL for eGFR 60—89 ml/min/1.73 m^2^. There were no J-shape relationships in low SUA levels.

Compared with previous studies, this study has some advantages. First, we performed the analysis using stratification of uric acid levels by 1 mg/dL. The mean and standard deviation of uric acid in this cohort is almost the same as the National Health and Nutrition Survey in Japan (mean 5.8 mg/dL, SD 1.3 mg/dL) [16] and is considered as a representative of the Japanese general population. Second, a large number of participants allowed subgroup analyses according to baseline kidney function.

Despite the fact that there were some studies which assessed SUA as a risk factor for kidney impairment [3, 17], no pharmacological management for asymptomatic hyperuricemia was addressed in the guidelines [18]. One of the reasons for this may be a lack in the definition of hyperuricemia from the viewpoint of CKD risk. For management of gout, hyperuricemia is defined as SUA 6.8 mg/dL, and patients are recommended to reduce their SUA to < 6 mg/dL [18]. On the other hand, a similar guide for kidney dysfunction is yet to be established. Previous studies [3, 17] showed higher risks of CKD in hyperuricemia, however, a definite threshold for SUA could not be reported because of small sample size.

Prior attempts to treat hyperuricemia for kidney protection have been successful. Allopurinol, one of the major drugs for hyperuricemia, was associated with increase in eGFR in patients with normal kidney function [17]. Same results were obtained in patients with CKD [19].

Regardless of case series on hypouricemia as a risk for kidney dysfunction [7], evidence has not been enough to decide on the role of low SUA levels. For mild hypouricemia, SUA < 4.0 mg/dL, risk for decreases in eGFR has been proved [4], but the precise risk for CKD is uncertain.

Linear increase in risk for CKD was consistent with previous study results [3]; further, we provided a clear SUA threshold for CKD risk. A SUA threshold of 6.0 mg/dL for normal kidney function was consistent with guidelines for the prevention of gout [18]. Interestingly, it is also similar to the cardiovascular risk threshold [20], as reported in the first National Health and Nutrition Examination Survey (NHANES I). Unique micro- and macrovascular thresholds are common for other risk factors such as blood pressure.[21] Guidelines for the management of high blood pressure recommend goals of treatment regardless of health outcomes such as overall mortality, cardiovascular disease-related mortality, and CKD-related mortality.

SUA thresholds as significant risk factors were different in subgroups according to baseline kidney function. In participants with mild kidney function, a slight increase in SUA (> 6.0 mg/dL) was a significant risk factor for kidney impairment. There is no clear answer for these facts, but like other common risk factors, such as hypertension for stroke [22], impact of hyperuricemia for CKD may vary according to a patient’s kidney status. In a previous report, people with eGFR 60–89 ml/min/1.73 m^2^ presented with lowest decline in eGFR [23] and were also considered to be low-risk groups for cardiovascular diseases [24]. This category in the absence of other markers, such as proteinuria, does not constitute CKD [25]. Regarding the impact of mild kidney dysfunction, recent studies revealed its growing importance in combination with specific risk factors. For example, mild kidney dysfunction with chest pain was a predictor of cardiovascular and all-cause mortality.[10] Taking into account the impact of uric acid, strategies to prevent loss of kidney function for patients with mild kidney dysfunction may be different for patients with normal kidney function.

Very low SUA levels were thought to be risk factors for acute renal failure [7], and may be associated with similar results of a previous Japanese cohort study showed risk of low SUA levels.[4] In this study, low SUA level was not a significant risk factor for kidney insufficiency. No J-shape relationship was observed but slight positive risk was found subgroups with eGFR 60–89 ml/min/1.73 m^2^. One possible reason is the relatively higher age in our cohort. Reported median age of acute renal failure was 17 years [26] and the mean age of cohorts with J-shape was 40 years [4]. To confirm the positive risks of low SUA for CKD, cohort studies on younger age patients may be required.

This study has several limitations. First was that we adopted an eGFR of < 60 ml/min/1.73 m^2^ and slopes of eGFR as surrogate outcomes of end-stage kidney disease. Second, this study enrolled only subjects with baseline and follow-up data, which may have led to some bias. For example, blood tests may have not been conducted for participants with low risk of disease so they were not included in the study. Third, we were unable to include the confounding factors of alcohol consumption, medication use, and status of metabolic syndrome. It is a well-known fact that alcohol consumption and diuretic use are closely correlated with hyperuricemia and some classes of antihypertensive agents may directly affect kidney function. Lacking information of metabolic syndrome, which may play a causal role for kidney dysfunction, is limitation in this study. There was a possibility that these factors were not evenly distributed across the participants by SUA levels, and they might explain the relationships between hyperuricemia and kidney dysfunction to some extent. Fourth, we could not consider treatment of diabetes or a self-reported history of diabetes as the definition of diabetes. It might result in underestimation of diabetes in this study.

In conclusion, high SUA was found to be risk factor for kidney impairment in maels. In participants with mild kidney dysfunction, slight elevation of SUA levels (≥ 6.0 mg/dL) posed a significant risk factor for CKD and rapid decline in eGFR. Studies on younger cohorts and females with high SUA levels will be required to clear the effect of low SUA levels on CKD. To more precisely examine and confirm the effect of SUA, external validation in cohorts and information regarding the effects of alcohol consumption and drugs on SUA levels will be required.

## Supporting Information

S1 Fig Relationships of uric acid and kidney dysfunction by gender and baseline kidney function. Adjusted for baseline age, systolic blood pressure, body mass index, hemoglobin, HDL cholesterol, triglyceride, urinary protein, and diabetes.
(EPS)
